# Molecular Research in Medical Genetics

**DOI:** 10.3390/ijms23126625

**Published:** 2022-06-14

**Authors:** Emanuela Viggiano

**Affiliations:** Department of Prevention, Hygiene and Public Health Service, ASL Roma 2, 00157 Rome, Italy; emanuela.viggiano@aslroma2.it

About 19,000–20,000 protein-coding genes in the human genome have been identified [1]. Mutations in these genes can determine Mendelian disease or interact with other factors, such as the environment, to determine complex diseases.

The pattern of inheritance is very often simple to recognize in Mendelian diseases, but the phenotype is not always predictable due to several mechanisms such as epigenetic modification.

An example of an epigenetic mechanism is genomic imprinting, which is the expression of a gene in a parent-of-origin–dependent manner. Imprinting alteration causes genetic disorders such as Beckwith Wiedemann, Prader–Willi, Angelman syndromes, and others [2,3].

Another important epigenetic mechanism is the X Chromosome Inactivation (XCI), in which there is a random inactivation of one X chromosome in all cells of females to preserve gene dosage.

Random XCI indicates that about 50% of the cells present the inactivation of one X chromosome; however, in some cases, females can exhibit skewed XCI, which is the preferential inactivation of one X chromosome, reported from several studies of more than 75%. The preferential inactivation of one X chromosome of more than 90% is indicated as extremely skewed XCI.

The role of skewed XCI in the phenotype of X-linked carriers is demonstrated in several diseases such as Duchenne/Becher disease [4,5], Haemophilia [6,7], and Lesch-Nyhan disease [8]. However, in other diseases, it is still debated [9,10,11]. 

The phenotype is also related to the type of mutations, and many studies are on genotype/phenotype correlation.

In particular, in metabolic diseases, mutations in the gene associated with the disease that causes the loss of the protein correlate with a severe phenotype, while mutations that decrease the level of the protein determine a less severe phenotype depending on the level of protein expression [12].

Research in Medical Genetics is an important target for finding the causes, inheritance, and treatment of genetic disorders. In particular, the discovery of new genes, pathogenic variants, and the mechanisms at the basis of phenotypes helps clinicians and researchers diagnose and find a therapeutic approach.

Today, despite a large amount of research in medical genetics, the phenotype description of genetic disorders with a molecular basis known is reported for about 6300 genes, while for many other diseases, the pathogenic mechanisms remain unknown (https://www.omim.org/statistics/entry, accessed on 30 May 2022).

To this aim, in the last decades, very promising techniques called “new generation sequencing” have shown very important results.

Starting with clinical observation is possible to use these new approaches to find rapidly pathogenic variations. For example, Alesi et al. [13] report the clinical observation of a boy with a severe short stature, growth hormone deficiency, psychomotor delay, corpus callosum hypoplasia, low-grade glioma, spastic paraparesis, and osteoporosis, and they identify new homozygous variations in HESX1 and COL1A1 genes using an SNP-array analysis and exome sequencing.

Castilla-Vallmanya et al. [14] used a similar approach, starting from clinical observation of severe neurodevelopmental delay associated with dysmorphism in two first degree cousins, ending with the molecular analysis with Whole-Exome Sequencing (WES), discovering two de novo variants, one in PORCN, which is responsible for Goltz-Gorlin syndrome, and the other in ZIC2 associated with holoprosencephaly 5.

Another interesting approach for identifying genomic loci associated with diseases, including cancer predisposition, is the Genome-wide Association Studies (GWAS). For example, more than 170 loci were correlated with hereditary prostatic cancer [15], in addition to a mutation in BRCA and MMR genes using GWAS [16].

Over the years, we have also assisted in the evolution of gene therapy that has two principal targets: one is the overexpressing of a therapeutic gene with the delivery of transgene, and the other is the correction of the pathological mechanisms to produce functional gene. In the last few years, the use of gene-editing technologies has demonstrated promising results. In particular, the use of antisense oligonucleotides was useful to correct some point mutations in Duchenne Muscular Dystrophy [17,18] and Epidermolysis Bullosa [19].

This Special Issue on “Molecular Research in Medical Genetics” will cover a selection of very interesting recent research topics and current review articles related to molecular mechanisms in human genetic diseases.
